# Epidemiological Profile of Emergency Hospitalizations and In-Hospital Deaths Due to Acute Pancreatitis and Other Pancreatic Diseases in Brazil, 2015-2024

**DOI:** 10.7759/cureus.117823

**Published:** 2026-10-12

**Authors:** Flávia M Amorim, Gustavo S Wurzius, Hoto G Valani, Cesar Augusto G Banhatto Vasconcellos, Mateus F Marques, Tuany C Bernardi, Lucas F Ananias

**Affiliations:** 1 General Medicine, Universidade Anhembi Morumbi, São Paulo, BRA; 2 General Medicine, Faculdade Morgana Potrich, Mineiros, BRA; 3 General Medicine, Universidade do Sul de Santa Catarina, Palhoça, BRA; 4 General Medicine, Universidade Estácio de Sá, Barbacena, BRA; 5 General Medicine, Centro Universitário São Camilo, São Paulo, BRA; 6 Medicine, Universidade Paranaense, Umuarama, BRA; 7 Infectious Disease, Universidade Federal do Triângulo Mineiro, Uberaba, BRA

**Keywords:** brazilian unified health system, hospitalization, hospital mortality, pancreatic diseases, pancreatitis

## Abstract

Background: Acute pancreatitis and other pancreatic diseases contribute to emergency hospital utilization in Brazil. Describing their temporal, regional, and demographic patterns may inform healthcare planning.

Objective: To analyze the epidemiological profile and temporal trends of Brazilian Unified Health System (SUS)-funded emergency admission records and in-hospital deaths under the combined International Statistical Classification of Diseases and Related Health Problems, 10th Revision (ICD-10) categories K85-K86 in Brazil between 2015 and 2024.

Methods: This nationwide ecological, retrospective time-series study used aggregated data from the Brazilian Unified Health System Hospital Information System (SIH/SUS). Approved Hospital Admission Authorizations (AIHs), rather than unique patients, were analyzed by processing year, hospital region, sex, and age group. Indicators included absolute counts, crude and stratum-specific population-based rates per 100,000 inhabitants, and recorded in-hospital deaths per 100 approved AIHs. Overall rates were not age-standardized. Trends in admission rates and AIH-based death percentages were assessed using the Joinpoint Regression Program, with annual percentage change and average annual percentage change (AAPC) estimates. Statistical significance was set at p < 0.05.

Results: Overall, 333,438 approved AIHs and 16,623 in-hospital deaths were recorded. The national admission rate increased from 14.82 to 17.11 per 100,000 inhabitants (AAPC: 1.80%; p < 0.05). Admission counts decreased in 2021 and subsequently increased, peaking in 2023. The Southeast accounted for the largest absolute numbers of admissions and deaths, whereas the South had the highest crude population-based rates. The North and Northeast showed the largest average annual increases in admission rates. Men accounted for most admissions and deaths, although their proportional contribution decreased. Admissions were concentrated among adults aged 30-69 years, while deaths were concentrated among older adults. Annual death counts remained relatively stable, whereas recorded deaths per 100 approved AIHs decreased from 5.67 to 4.63 (AAPC: -2.24%; p < 0.001).

Conclusion: Within SIH/SUS, emergency admission rates under K85-K86 increased, while recorded deaths relative to approved AIHs declined. Regional and demographic differences may inform surveillance and assessment of healthcare needs. These findings describe administrative hospital utilization and in-hospital outcomes and do not establish changes in disease incidence or improvements in care.

## Introduction

Pancreatic diseases comprise a heterogeneous spectrum that includes acute pancreatitis, recurrent acute pancreatitis, chronic pancreatitis, and other inflammatory or structural disorders. Acute pancreatitis is characterized by an abrupt inflammatory process and an unpredictable clinical course. Although most episodes are self-limited, persistent organ failure and infected pancreatic or peripancreatic necrosis may result in substantial morbidity and mortality [1]. Moreover, recurrent disease, chronic pancreatitis, exocrine insufficiency, and post-pancreatitis diabetes extend its burden beyond the initial hospitalization [2].

The epidemiology of pancreatitis varies considerably across populations. A systematic review and meta-analysis of 44 population-based studies found that the incidence of acute pancreatitis increased by an average of 3.07% per year, with particularly consistent growth in North America and Europe [3]. Global estimates also indicate that the absolute numbers of cases and deaths have risen despite reductions in several age-standardized rates, reflecting the combined influence of population growth, aging, and socioeconomic inequalities [4]. Gallstones and alcohol remain the predominant etiologies worldwide, but their relative contributions vary geographically and according to sex and age [5]. Advanced age and comorbidity are also strongly associated with severe disease and death, making demographic transition relevant to hospital burden and outcomes [6].

In Brazil, analyses of the Hospital Information System of the Unified Health System have demonstrated increasing hospital utilization related to pancreatic diseases. Acute pancreatitis hospitalizations increased by 42.8% between 2008 and 2016 [7], while a subsequent study covering 2016-2020 reported continued growth and important regional, age, and sex differences [8]. However, nationwide evidence extending through the COVID-19 pandemic and subsequent years remains limited, particularly regarding emergency admission patterns and associated in-hospital outcomes across geographic and demographic groups.

To address this gap, this study aimed to characterize the epidemiological profile and temporal patterns of Brazilian Unified Health System (SUS)-funded emergency admission records and in-hospital deaths under the combined International Statistical Classification of Diseases and Related Health Problems, 10th Revision (ICD-10) categories K85-K86, comprising acute pancreatitis and other pancreatic diseases, in Brazil from 2015 to 2024. Specifically, it sought to describe approved Hospital Admission Authorizations (AIHs) and recorded deaths by geographic region, sex, and age group; calculate corresponding population-based admission and in-hospital mortality rates; and assess temporal trends in admission rates and recorded in-hospital deaths relative to approved AIHs.

## Materials and methods

Study design and data sources

This nationwide ecological, retrospective time-series study examined SUS-funded emergency hospital admissions and recorded in-hospital deaths under the combined ICD-10 categories K85-K86, comprising acute pancreatitis and other pancreatic diseases, in Brazil from 2015 to 2024. Data were obtained from the Hospital Information System of the Brazilian Unified Health System (SIH/SUS), accessed through the TABNET (DATASUS, Ministry of Health of Brazil, Brasília, Brazil) platform of the Department of Informatics of the Unified Health System (DATASUS) [9]. The initial data extraction was performed in May 2026.

The hospital database was accessed through “Informações de Saúde (TABNET),” “Epidemiológicas e Morbidade,” and “Morbidade Hospitalar do SUS (SIH/SUS),” selecting “Geral, por local de internação, a partir de 2008.” The analysis covered Brazil and its five geographic regions: North, Northeast, Southeast, South, and Central-West. Geographic classification referred to the location of the hospital, rather than the patient’s place of residence.

The diagnostic selection was “Pancreatite aguda e outras doenças do pâncreas” under “Lista Morb CID-10,” corresponding to the combined categories K85-K86. The study period comprised processing years 2015-2024. Hospital counts were extracted using “AIH aprovadas,” and in-hospital deaths were obtained using “Óbitos.” Emergency admissions were defined by selecting “Urgência” under “Caráter de atendimento”.

For regional age-stratified extractions, “Faixa Etária 1” was selected as the row variable and “Ano processamento” as the column variable. Queries were repeated for each region and for both content measures. Additional tabulations provided annual national and regional totals and distributions by sex. Because the temporal variable was processing year, annual counts represented the administrative processing period and did not necessarily correspond to the year of admission.

SIH/SUS includes admissions financed by SUS in public hospitals and contracted private institutions. Admissions funded exclusively through private insurance or out-of-pocket payments are not captured. The counting unit was an approved Hospital Admission Authorization (Autorização de Internação Hospitalar; AIH), rather than a unique patient. Approved AIHs may include authorizations for prolonged stays, and the aggregated data did not permit identification of repeated authorizations or readmissions involving the same individual.

Annual population denominators were obtained from the TABNET database “Resident population: study of population estimates by municipality, age, and sex, 2000-2025,” using estimates made available by the Brazilian Ministry of Health based on demographic data from the Brazilian Institute of Geography and Statistics (IBGE) [10]. Population tables used “População residente” as the content measure and “Ano” as the column variable, selecting 2015-2024. Demographic and regional selections corresponded to those of the hospital tabulations. For age-specific denominators, “Faixa Etária 1” was used as the row variable.

Variables and indicators

The variables analyzed included processing year, geographic region of hospitalization, sex, age group, number of approved AIHs, and number of recorded in-hospital deaths. Age groups were defined as 0-4, 5-9, 10-14, 15-19, 20-29, 30-39, 40-49, 50-59, 60-69, 70-79, and 80 years or older. Hospital records classified as younger than one year and 1-4 years were combined to form the 0-4-year category, matching the corresponding population denominator.

Annual AIH-based hospitalization rates were calculated by dividing the number of approved AIHs by the corresponding resident population and multiplying the result by 100,000. Numerators and denominators were matched by year and by the geographic and demographic categories used in each analysis. Thus, regional rates used the population of the corresponding region, sex-specific rates used the population of the respective sex, and age-specific rates used the population of the corresponding age group. Regional age-specific rates matched the age group, region, and year.

These indicators were crude or stratum-specific population-based rates. Calculating rates within age groups did not constitute age standardization using a common reference population. Because hospital events were classified by hospital location, regional rates described recorded service utilization relative to the resident population and should not be interpreted as hospitalization risks among residents of each region.

Mortality was assessed using two indicators. Population-based in-hospital mortality rates were calculated by dividing recorded in-hospital deaths by the corresponding annual resident population and multiplying the result by 100,000. These rates were calculated for Brazil and its regions and according to sex and age group, matching the relevant population denominators.

The AIH-based in-hospital death indicator was calculated by dividing recorded in-hospital deaths by the corresponding number of approved AIHs and multiplying the result by 100. This percentage represented recorded deaths relative to approved administrative authorizations, rather than case fatality among unique patients. It was distinguished from population-based mortality rates and from the predefined TABNET mortality indicator, which uses AIHs counted as hospitalizations as its denominator. All mortality measures were restricted to deaths recorded during the included SUS-funded hospital admissions. Deaths occurring before admission or after discharge were unavailable; therefore, these indicators did not estimate total mortality from pancreatic diseases or longer-term survival.

Statistical analysis

Temporal trends were assessed using joinpoint regression for two outcomes: annual AIH-based emergency hospitalization rates per 100,000 inhabitants and the annual proportion of admissions resulting in in-hospital death. Separate models were fitted for Brazil and each of its five geographic regions, comprising six hospitalization-rate series and six admission-based mortality proportion series. Each series included 10 annual observations from 2015 to 2024.

Continuous piecewise log-linear models were fitted with calendar year as the explanatory variable and the natural logarithm of the corresponding annual rate or proportion as the response. Candidate models allowed zero or one joinpoint, with at least three annual observations in each segment, including the joinpoint year. For a model containing one joinpoint, this required at least two observations before and two after the change point. The final model was selected using the Bayesian information criterion (BIC).

For each segment, the annual percentage change (APC) was calculated as 100 × [exp(β) - 1], where β represents the estimated annual slope on the logarithmic scale. The average annual percentage change (AAPC) summarized the entire 2015-2024 interval by weighting the segment-specific slopes according to their duration in years, using AAPC = 100 × {exp[Σ(dᵢβᵢ)/Σdᵢ] - 1}. Segment durations totaled nine elapsed years across the 10 annual observations. When no joinpoint was selected, the APC and AAPC were numerically identical.

Estimates were reported with 95% confidence intervals and two-sided p-values, adopting a significance level of 0.05. Positive and negative estimates with p < 0.05 were classified as increasing and decreasing trends, respectively. Otherwise, the findings were described as showing no statistically significant temporal change. For the proportion of admissions resulting in death, APC and AAPC represented relative annual percentage changes rather than changes in percentage points. Population-based in-hospital mortality rates and demographic distributions were summarized descriptively. These analyses were distinguished from the joinpoint models fitted to AIHs rates and admission-based mortality proportions.

Ethical considerations

The study exclusively used publicly available, aggregated, and anonymized secondary data, without individual identifiers or direct contact with patients. Therefore, informed consent and review by a Research Ethics Committee were not required, in accordance with Brazilian National Health Council Resolution No. 510/2016 and applicable guidance from the Brazilian Research Ethics Committee/National Research Ethics Commission system. The ethical principles established by National Health Council Resolution No. 466/2012 were observed.

## Results

Hospitalizations

Between 2015 and 2024, the Brazilian Unified Health System recorded 333,438 AIH-based emergency admissions for acute pancreatitis and other pancreatic diseases. These figures represent admission records based on approved Hospital Admission Authorizations (AIHs), rather than unique individual patients, and correspond to an annual mean of 33,344 admissions. The annual number increased from 30,005 in 2015 to 36,382 in 2024, representing a cumulative increase of 21.3% and a compound annual growth rate of 2.16%. The population-based admission rate increased by 15.4%, from 14.82 to 17.11 per 100,000 inhabitants. Admission counts increased progressively from 2015 to 2020, when 33,692 admissions were recorded, followed by a 1.8% reduction to 33,069 in 2021. The largest year-to-year increase occurred in 2022, when admissions increased by 8.6% to 35,902. Both the highest annual count and the highest population-based admission rate were observed in 2023, with 36,876 admissions and 17.42 per 100,000 inhabitants. In 2024, the admission count decreased by 1.3% compared with the previous year.

At the national level, joinpoint regression identified a significant increase in emergency hospitalization rates throughout the study period, with an APC and AAPC of 1.80% (p < 0.05). Regional differences in temporal trends were observed (Figure 1). In the North, hospitalization rates increased significantly between 2015 and 2022 (APC = 5.81%; p < 0.05), followed by a nonsignificant decline between 2022 and 2024 (APC = -3.73%). Despite the change in the final segment, the overall trend remained significantly increasing, with an AAPC of 3.61% (p < 0.05). Similarly, the Northeast showed a significant increase from 2015 to 2022 (APC = 4.02%; p < 0.05), followed by a stable trend from 2022 to 2024 (APC = 0.32%; p ≥ 0.05), resulting in a significant AAPC of 3.19% (p < 0.05). No joinpoints were detected in the Southeast, South, or Central-West, which presented single increasing trends throughout 2015-2024, with APCs of 0.65%, 2.22%, and 2.67%, respectively. The increases were statistically significant in the South and Central-West (p < 0.05) but not in the Southeast.

**Figure 1:**
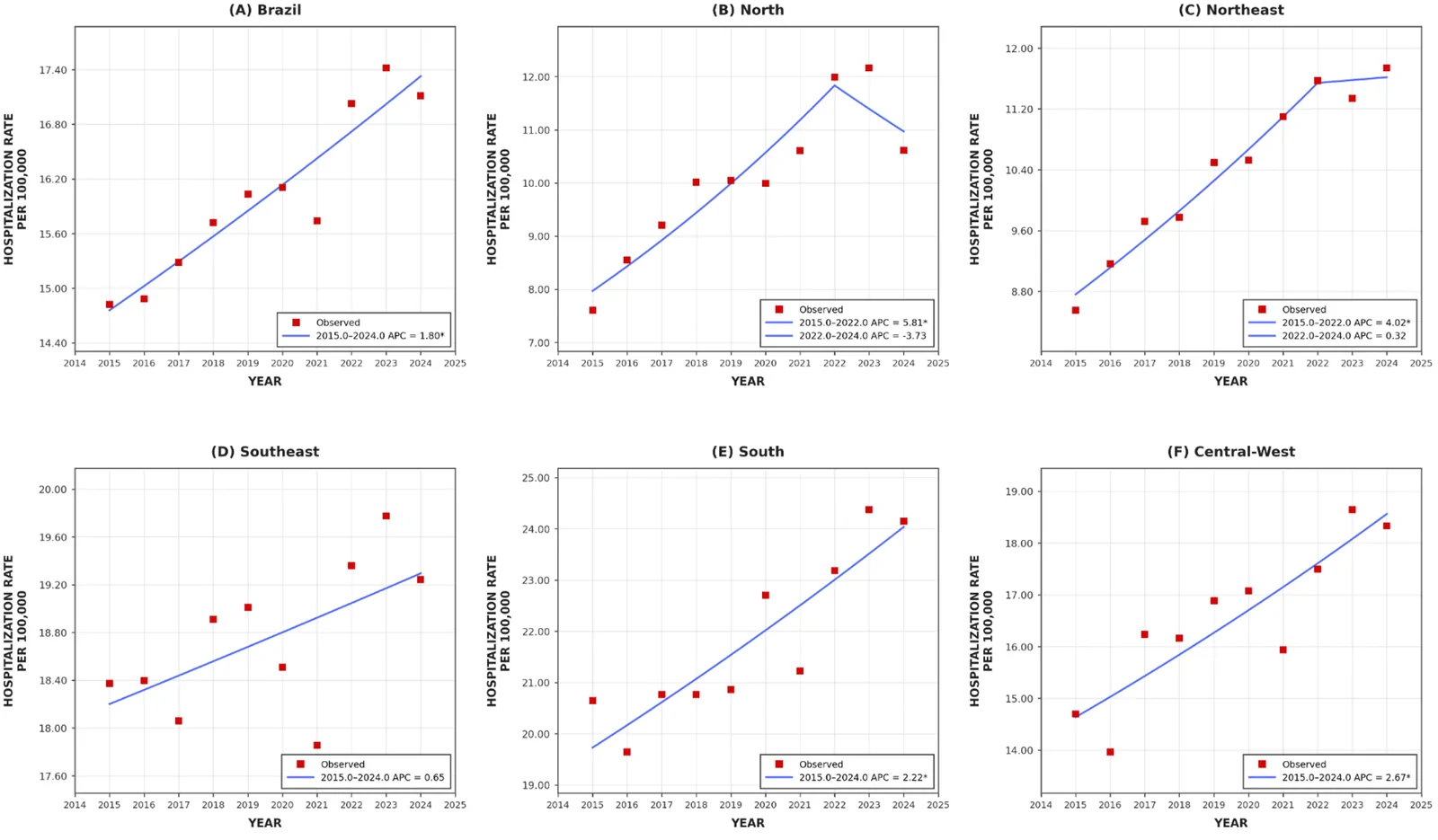
Joinpoint regression analysis of emergency hospitalization rates per 100,000 inhabitants for acute pancreatitis and other pancreatic diseases in Brazil and its macroregions, 2015-2024. Prepared by the authors using data from SIH/DATASUS, with the Joinpoint Regression Program v6.0.1. (A) Brazil; (B) North; (C) Northeast; (D) Southeast; (E) South; and (F) Central-West. Red squares represent the observed annual rates, and blue lines represent the fitted log-linear trends. An asterisk indicates statistical significance at p < 0.05. Panel-specific y-axis scales were used to improve visualization. SIH: Hospital Information System, DATASUS: Department of Informatics of the Unified Health System, APC: annual percentage change.

Marked regional differences were also observed in the absolute and relative burden of hospitalizations. The Southeast accounted for 163,775 hospitalizations, representing 49.1% of the national total, followed by the South with 65,887 (19.8%), Northeast with 58,589 (17.6%), Central-West with 26,983 (8.1%), and North with 18,204 (5.5%). Although the Southeast remained responsible for the greatest absolute number, its contribution to national hospitalizations decreased from 52.4% in 2015 to 46.9% in 2024. During the same period, the contributions of the Northeast, South, Central-West, and North increased from 15.7% to 18.4%, 20.0% to 20.7%, 7.5% to 8.6%, and 4.4% to 5.4%, respectively.

Hospitalizations increased in all five regions between 2015 and 2024. The largest cumulative increase occurred in the North, where admissions rose from 1,311 to 1,982 (51.2%; compound annual growth rate, 4.70%). This was followed by the Northeast, from 4,721 to 6,705 (42.0%; 3.98% annually); Central-West, from 2,265 to 3,130 (38.2%; 3.66% annually); South, from 6,000 to 7,513 (25.2%; 2.53% annually); and Southeast, from 15,708 to 17,052 (8.6%; 0.92% annually). The South consistently had the highest population-based hospitalization rate, increasing from 20.65 per 100,000 inhabitants in 2015 to 24.15 in 2024. Over the same period, hospitalization rates increased from 18.37 to 19.24 in the Southeast, 14.70 to 18.33 in the Central-West, 8.55 to 11.74 in the Northeast, and 7.61 to 10.62 per 100,000 in the North.

Men accounted for 177,177 hospitalizations (53.1%), compared with 156,261 (46.9%) among women. Male predominance was observed throughout the series, although the male proportion decreased from 55.4% in 2015 to 52.1% in 2024. Female hospitalizations increased by 30.3%, from 13,376 to 17,423, whereas male hospitalizations increased by 14.0%, from 16,629 to 18,959. The hospitalization rate remained higher among men but increased more markedly among women. Between 2015 and 2024, the rate increased from 16.81 to 18.29 per 100,000 men and from 12.93 to 16.00 per 100,000 women, indicating a reduction in the magnitude of the sex-related difference over time (Figure 2).

**Figure 2:**
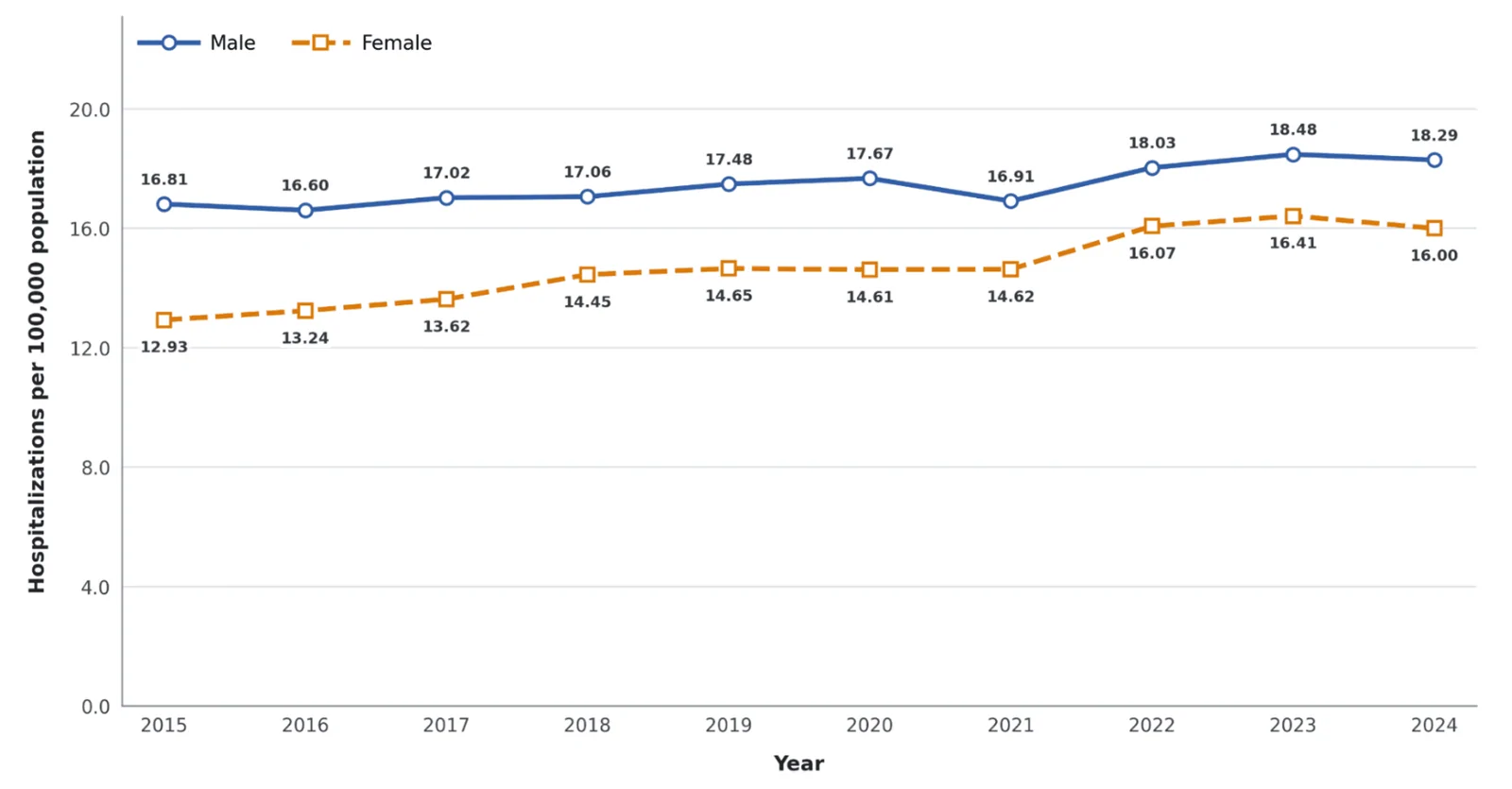
Temporal evolution of annual emergency hospitalization rates for acute pancreatitis and other pancreatic diseases according to sex, Brazil, 2015-2024. Prepared by the authors using data from SIH/DATASUS and IBGE. Rates are expressed per 100,000 inhabitants and were calculated using annual sex-specific population estimates. SIH: Hospital Information System, DATASUS: Department of Informatics of the Unified Health System, IBGE: Brazilian Institute of Geography and Statistics.

Hospitalizations were concentrated among adults aged 30-69 years, who accounted for 234,600 admissions (70.4% of the total). The largest number occurred among individuals aged 40-49 years (n = 66,576; 20.0%), followed by those aged 50-59 years (n = 60,487; 18.1%), 30-39 years (n = 59,854; 18.0%), and 60-69 years (n = 47,683; 14.3%). Overall, individuals aged ≥60 years accounted for 99,329 admissions (29.8%), including 32,279 among those aged 70-79 years and 19,367 among those aged ≥80 years. Between 2015 and 2024, hospitalizations increased by 33.1%, 31.5%, and 26.9% in the 60-69, 70-79, and ≥80-year age groups, respectively, resulting in an increase from 8,651 to 11,363 admissions among older adults. Among individuals aged ≥30 years, hospitalization rates increased progressively with advancing age (Figure 3). In 2024, rates ranged from 19.13 per 100,000 population among those aged 30-39 years to 46.26 per 100,000 among those aged ≥80 years, with intermediate values of 23.30, 25.72, 28.74, and 35.46 per 100,000 in the 40-49, 50-59, 60-69, and 70-79-year age groups, respectively.

**Figure 3:**
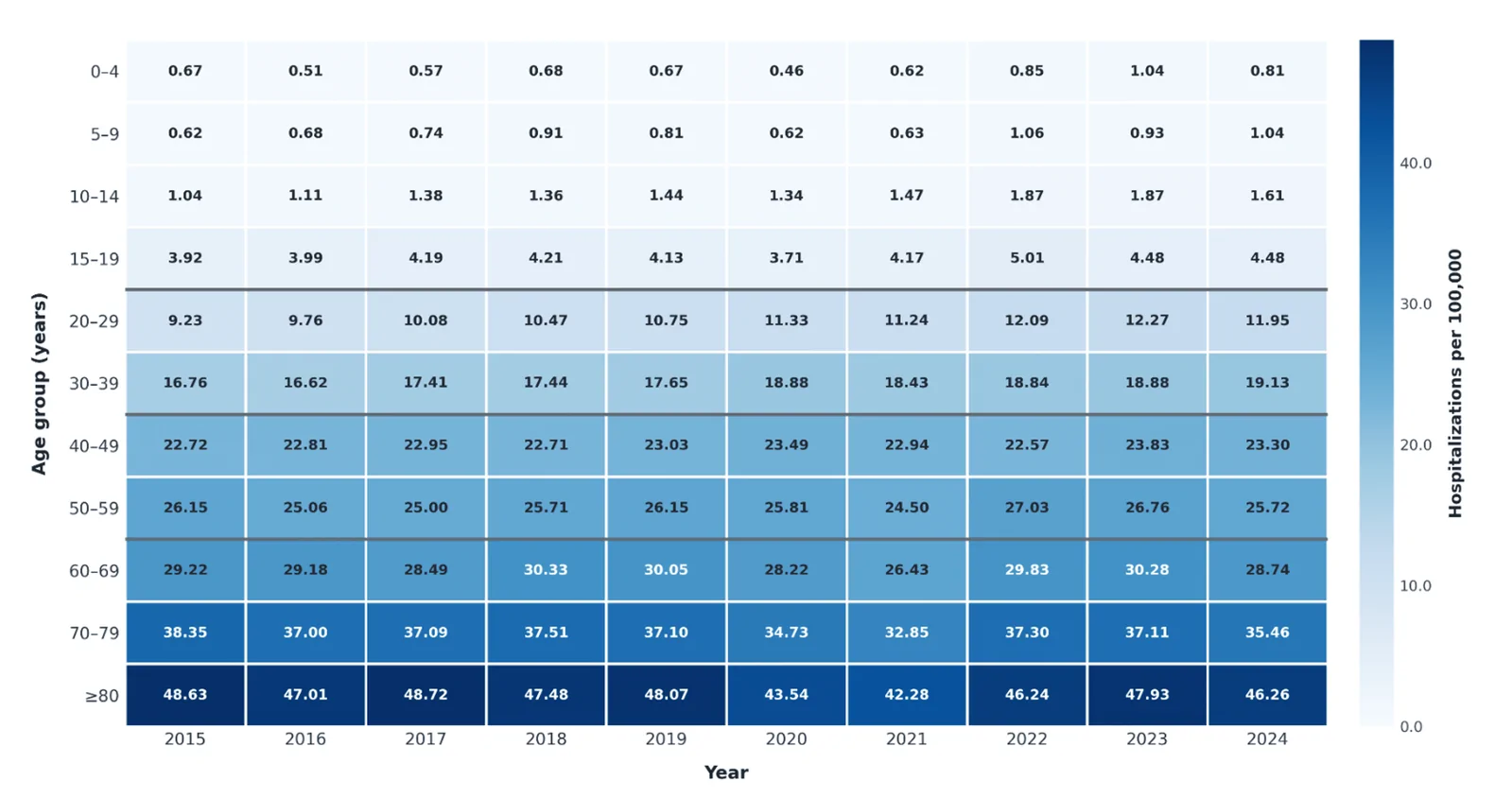
Annual emergency hospitalization rates for acute pancreatitis and other pancreatic diseases according to age group and year, Brazil, 2015-2024. Prepared by the authors using data from SIH/DATASUS. Rates are expressed per 100,000 age-specific population. Exact annual values are shown in each cell, and darker shades represent higher rates. SIH: Hospital Information System, DATASUS: Department of Informatics of the Unified Health System.

Regional analyses revealed differences in age-specific AIHs rates and their changes between 2015 and 2024. The South consistently recorded the highest rates among individuals aged ≥50 years throughout the study period. In 2024, it also presented the highest rates in every age group ≥30 years. Among individuals aged ≥80 years, rates reached 77.79 per 100,000 population in the South, followed by the Southeast (50.55), Central-West (46.53), North (39.43), and Northeast (27.88). The rate in the South was 2.79 times that observed in the Northeast. Regional differences were also evident among individuals aged 70-79 years, with rates ranging from 24.17 in the Northeast to 59.29 in the South, and among those aged 60-69 years, with corresponding values of 20.35 and 42.55 per 100,000 population.

Changes between the first and last study years differed across age groups and regions. Among individuals aged 30-39 years, rates increased by 41.7% in the Northeast, 32.9% in the South, 26.8% in the Central-West, and 19.8% in the North, whereas the Southeast recorded a 2.2% decrease. Among those aged 40-49 years, increases were observed in the Northeast (28.0%), Central-West (19.9%), North (16.7%), and South (4.4%), while the Southeast recorded a 5.9% reduction. In the Southeast, rates decreased between 2015 and 2024 in all age groups ≥30 years, with the largest relative reduction among individuals aged 70-79 years (14.3%).

Among older adults, the North recorded increases of 25.9% and 12.2% in the 60-69 and 70-79-year age groups, respectively. However, rates among individuals aged ≥80 years decreased in the Central-West (18.8%), South (8.1%), North (7.2%), and Southeast (4.2%), while increasing by 2.1% in the Northeast. The highest age-specific rate observed across all regions and years was 84.68 per 100,000 population among individuals aged ≥80 years in the South in 2015.

In-hospital deaths

Among 333,438 AIH-based emergency admissions, representing admission records rather than unique individual patients, 16,623 in-hospital deaths were recorded, corresponding to an annual mean of 1,662 deaths and an overall ratio of 4.99 deaths per 100 approved AIHs. Annual deaths remained within a relatively narrow range, varying from 1,593 in 2016 to 1,728 in 2020. Between 2015 and 2024, deaths decreased by 1.0%, from 1,701 to 1,684, while admissions increased by 21.3%, from 30,005 to 36,382. Together, these changes corresponded to a decline in the AIH-based in-hospital death proportion from 5.67% to 4.63%, indicating fewer recorded deaths relative to admission volume. After peaking in 2020, deaths decreased by 5.5% in 2021 and by a further 1.2% in 2022, when 1,613 deaths were recorded. Subsequent increases of 3.3% in 2023 and 1.0% in 2024 did not bring the annual count above its 2015 level. The population-based in-hospital mortality rate also decreased over the study period, falling by 5.7%, from 0.84 to 0.79 per 100,000 inhabitants.

The cumulative hospital case-fatality rate was highest in the Northeast (5.59%), followed by the South (5.08%), Southeast (4.82%), Central-West (4.67%), and North (4.58%). Nationally, annual hospital case fatality decreased from 5.67% in 2015 to 4.63% in 2024, representing an absolute reduction of 1.04 percentage points and a relative reduction of 18.4%. The lowest value was observed in 2022 at 4.49%, followed by modest increases to 4.52% in 2023 and 4.63% in 2024.

Joinpoint regression confirmed a significant national decline in hospital case fatality throughout the study period (APC and AAPC = -2.24%; 95% CI: -3.01 to -1.47; p < 0.001). Significant declining trends were also observed in the North (APC and AAPC = -5.43%; 95% CI: -8.93 to -1.80; p = 0.009), Southeast (APC and AAPC = -1.99%; 95% CI: -3.24 to -0.72; p = 0.007), and Central-West (APC and AAPC = -4.88%; 95% CI: -6.65 to -3.07; p < 0.001). In the Northeast, a joinpoint was identified in 2017, with a significant decline between 2015 and 2017 (APC = -9.31%; 95% CI: -15.38 to -2.81; p = 0.012), followed by a nonsignificant reduction between 2017 and 2024 (APC = -1.34%; 95% CI: -3.08 to 0.44; p = 0.118). Despite the nonsignificant trend in the final segment, the Northeast had a significant overall reduction, with an AAPC of -3.17% (95% CI: -4.54 to -1.78; p = 0.001). Conversely, no significant temporal change was detected in the South (APC and AAPC = -1.00%; 95% CI: -2.43 to 0.45; p = 0.150) (Figure 4).

**Figure 4:**
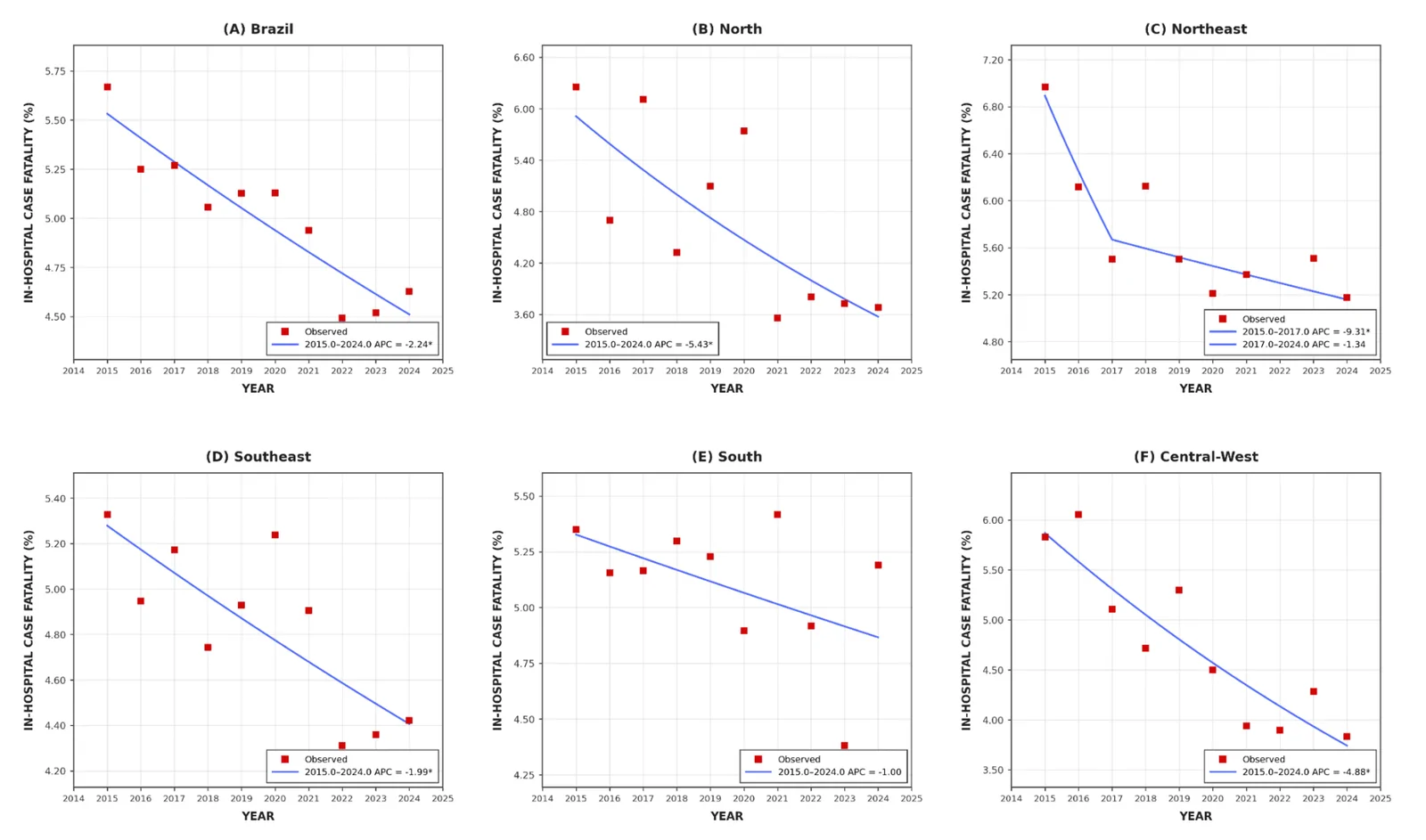
Joinpoint regression analysis of annual in-hospital case-fatality rates for emergency hospitalizations due to acute pancreatitis and other pancreatic diseases in Brazil and its macroregions, 2015-2024. Prepared by the authors using data from SIH/DATASUS, with the Joinpoint Regression Program v6.0.1. (A) Brazil; (B) North; (C) Northeast; (D) Southeast; (E) South; and (F) Central-West. An asterisk indicates statistical significance at p < 0.05. Panel-specific y-axis scales were used to improve visualization. SIH: Hospital Information System, DATASUS: Department of Informatics of the Unified Health System, APC: annual percentage change.

The Southeast accounted for the largest absolute number of deaths, with 7,901 events (47.5%), followed by the South with 3,349 (20.1%), Northeast with 3,278 (19.7%), Central-West with 1,261 (7.6%), and North with 834 (5.0%). The Southeast’s contribution to national deaths decreased from 49.2% in 2015 to 44.8% in 2024. Conversely, the contribution of the South increased from 18.9% to 23.2%, while that of the Northeast increased from 19.3% to 20.6%. In 2024, the North and Central-West accounted for 4.3% and 7.1% of national deaths, respectively.

Between 2015 and 2024, deaths increased in the South from 321 to 390 (21.5%) and in the Northeast from 329 to 347 (5.5%). Reductions occurred in the North, from 82 to 73 (11.0%); Southeast, from 837 to 754 (9.9%); and Central-West, from 132 to 120 (9.1%). The South had the highest population-based mortality rate in every year, increasing from 1.10 per 100,000 inhabitants in 2015 to 1.25 in 2024. In 2024, the mortality rates in the remaining regions were 0.85 in the Southeast, 0.70 in the Central-West, 0.61 in the Northeast, and 0.39 per 100,000 in the North. Descriptively, hospital case fatality decreased from 6.25% to 3.68% in the North, 6.97% to 5.18% in the Northeast, 5.33% to 4.42% in the Southeast, and 5.83% to 3.83% in the Central-West. The South presented only a modest reduction, from 5.35% in 2015 to 5.19% in 2024, consistent with the absence of a statistically significant Joinpoint trend in this region.

Men accounted for 9,719 deaths (58.5%), compared with 6,904 deaths (41.5%) among women. Male deaths exceeded female deaths in every year, although the male proportion decreased from 60.0% in 2015 to 56.7% in 2024. The number of male deaths decreased by 6.6%, from 1,021 to 954, whereas female deaths increased by 7.4%, from 680 to 730. The male mortality rate decreased from 1.03 to 0.92 per 100,000, while the female rate remained comparatively stable, changing from 0.66 to 0.67 per 100,000. The cumulative hospital case-fatality rate was higher among men than women (5.49% versus 4.42%). Between 2015 and 2024, hospital case fatality decreased from 6.14% to 5.03% among men and from 5.08% to 4.19% among women (Figure 5).

**Figure 5:**
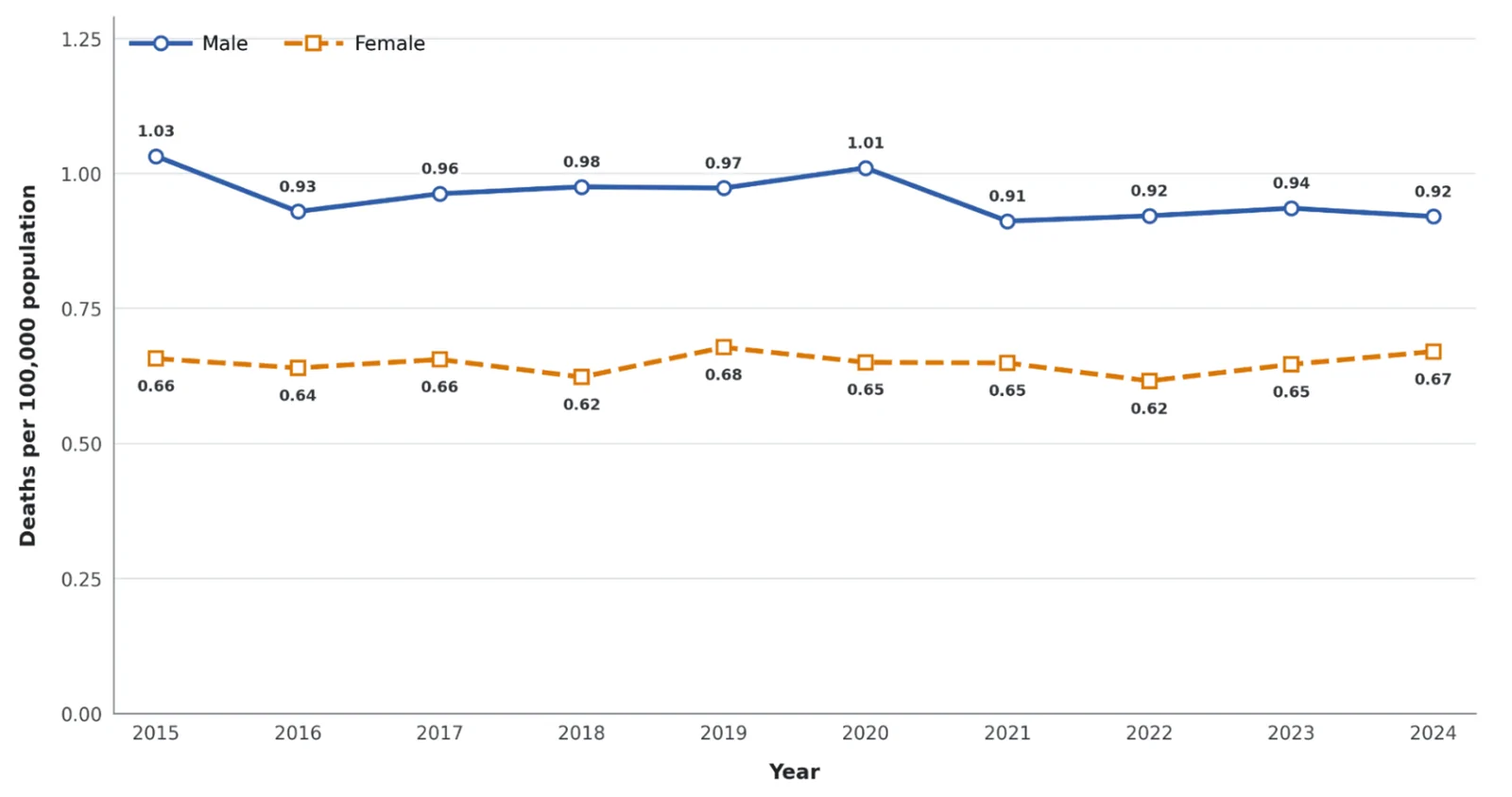
Temporal evolution of annual population-based mortality rates associated with emergency hospitalizations for acute pancreatitis and other pancreatic diseases according to sex, Brazil, 2015-2024. Prepared by the authors using data from SIH/DATASUS and IBGE. Rates are expressed per 100,000 inhabitants and were calculated using annual sex-specific population estimates. SIH: Hospital Information System, DATASUS: Department of Informatics of the Unified Health System, IBGE: Brazilian Institute of Geography and Statistics.

In-hospital deaths were disproportionately concentrated among older adults. The largest number occurred among those aged 60-69 years (n = 3,423; 19.9%), followed by those aged 70-79 years (n = 3,381; 19.7%) and ≥80 years (n = 3,100; 18.0%). Individuals aged 50-59 years accounted for 2,940 deaths (17.1%), whereas those aged 40-49, 30-39, and 20-29 years accounted for 2,277 (13.3%), 1,424 (8.3%), and 529 (3.1%), respectively. Only 105 deaths (0.6%) occurred among individuals aged <20 years. National age-specific rates increased progressively across successive adult age groups throughout the study period, remaining highest among individuals aged ≥80 years. Despite annual fluctuations, rates were lower in 2024 than in 2015 in all age groups aged ≥20 years. Among individuals aged ≥50 years, the largest relative reductions occurred in the 70-79-year (30.1%), 50-59-year (27.3%), and ≥80-year (25.3%) age groups, while the reduction among those aged 60-69 years was smaller (4.5%). The national crude population-based in-hospital death rate decreased by 5.6%, from 0.87 per 100,000 population in 2015 to 0.82 in 2024 (Figure 6).

**Figure 6:**
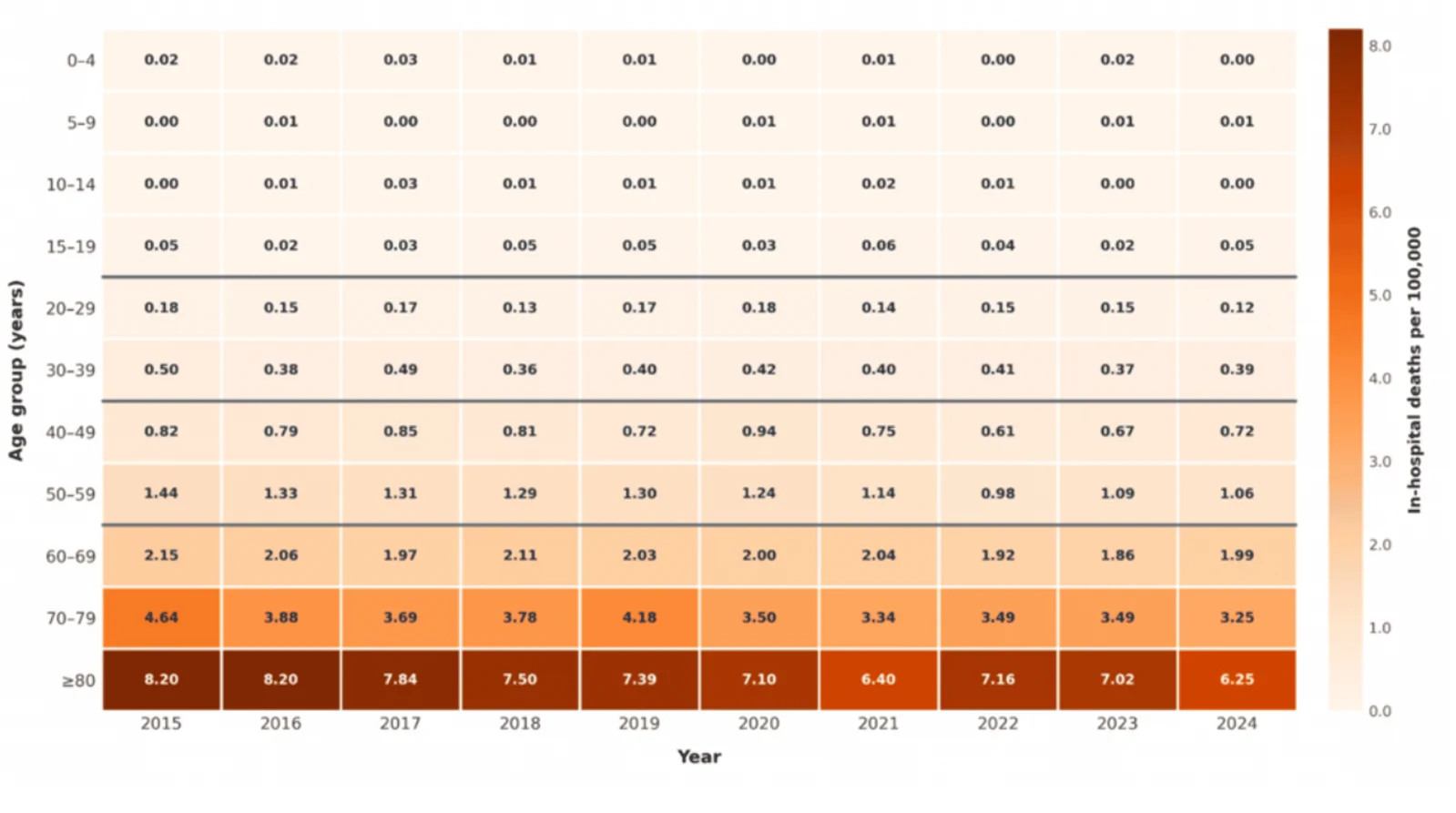
Age-specific population-based in-hospital death rates associated with acute pancreatitis and other pancreatic diseases in Brazil, 2015-2024. Prepared by the authors using data from SIH/DATASUS and IBGE population data available through DATASUS. Each cell presents the annual number of in-hospital deaths per 100,000 population in the corresponding age group; darker shades indicate higher rates. SIH: Hospital Information System, DATASUS: Department of Informatics of the Unified Health System, IBGE: Brazilian Institute of Geography and Statistics.

Regionally, the Southeast accounted for the largest proportion of deaths over the study period (n = 8,122; 47.3%), followed by the South (n = 3,447; 20.1%), Northeast (n = 3,422; 19.9%), Central-West (n = 1,272; 7.4%), and North (n = 916; 5.3%). In 2024, however, the highest crude population-based in-hospital death rate was observed in the South (1.28 per 100,000 population), followed by the Southeast (0.88), Central-West (0.71), Northeast (0.65), and North (0.43).

The concentration of deaths among older adults also varied across regions. Individuals aged ≥60 years accounted for 65.8% of deaths in the South (n = 2,268), 58.3% in the Southeast (n = 4,735), 52.3% in the Northeast (n = 1,788), 51.7% in the North (n = 474), and 50.2% in the Central-West (n = 639). Within each region, the largest cumulative number of deaths occurred among individuals aged 60-69 years in the Southeast (n = 1,644; 20.2%) and North (n = 186; 20.3%), 70-79 years in the Northeast (n = 634; 18.5%), ≥80 years in the South (n = 762; 22.1%), and 50-59 years in the Central-West (n = 237; 18.6%).

Regional differences were also observed in age-specific rates. In 2024, the South recorded the highest rates in every age group aged ≥40 years, reaching 1.66, 2.79, 5.79, and 11.09 per 100,000 population among individuals aged 50-59, 60-69, 70-79, and ≥80 years, respectively. Among individuals aged ≥80 years, rates in the remaining regions were 6.84 in the Central-West, 6.26 in the Southeast, 4.28 in the Northeast, and 3.25 in the North.

## Discussion

The increasing number of AIH-based emergency admissions for acute pancreatitis and other pancreatic diseases in Brazil extends previous evidence of a growing hospital burden within SUS-funded care [7,8]. International studies have also reported increasing incidence or hospitalization rates for pancreatitis, although these outcomes are not interchangeable with the administrative admission counts analyzed here [3,11-13]. The concurrent decline in recorded in-hospital deaths relative to approved AIHs parallels patterns described in large inpatient databases in the United States [12,13]. The observed regional heterogeneity, predominance of admissions among men, and concentration of deaths among older adults also align with previous Brazilian investigations and international evidence regarding geographical and age-related differences in pancreatic disease [4,6,8]. These findings describe patterns of hospital utilization and recorded in-hospital outcomes rather than individual disease incidence or mortality risk.

Previous Brazilian studies have documented increasing hospitalizations for nontraumatic abdominal emergencies and pancreatic diseases, suggesting that the present findings extend trends previously identified within the public healthcare system [7,8]. Internationally, a systematic review and meta-analysis demonstrated an increasing incidence of acute pancreatitis, while population-based research in Sweden and nationwide analyses in the United States reported comparable increases in incidence or hospitalization burden [3,11-13]. These patterns may reflect population aging, changes in exposure to biliary, alcohol-related, and metabolic risk factors, and variations in diagnostic recognition and healthcare utilization [2,3,5,11-13]. However, the present study could not distinguish the contributions of these mechanisms. Hospitalization records should not be interpreted as equivalent to incident cases because recurrent episodes, readmissions, and interhospital transfers may generate multiple records for the same individual [2,9,12,13]. Changes in admission thresholds, diagnostic practices, and administrative reporting may also influence recorded admission rates [9,12,13].

The temporary decline in admissions observed in 2021 occurred during the COVID-19 pandemic and is temporally consistent with changes in healthcare utilization reported for gastrointestinal conditions [14]. An analysis of inpatient data from California demonstrated reductions in hospitalizations for several gastrointestinal disorders, accompanied by increased in-hospital mortality among patients with acute pancreatitis [14]. Delayed presentation, altered healthcare-seeking behavior, and disruptions in routine healthcare delivery are possible explanations for such patterns, but these mechanisms were not measured in the present study [14]. Concomitant SARS-CoV-2 infection has also been associated with more severe acute pancreatitis, persistent organ failure, and increased short-term mortality [15]. Similarly, a nationwide Japanese survey documented changes in the frequency and management of acute pancreatitis during the pandemic [16]. Restoration of healthcare services and treatment of previously unmet demand are possible explanations for the subsequent increase in admissions in Brazil. Nevertheless, neither the pandemic’s contribution nor these proposed mechanisms can be established from the observed temporal sequence, and they should be regarded as hypotheses [14-16].

The concentration of hospitalizations and deaths in the Southeast is consistent with previous Brazilian studies and should be interpreted in relation to the region’s population size and concentration of healthcare services [7,8]. Absolute admission counts and population-based rates capture distinct dimensions of recorded hospital burden, and regional comparisons may be influenced by differences in demographic composition, healthcare infrastructure, referral patterns, and access to specialized care [4,8]. However, the age-specific analyses showed that in 2024, the South had the highest hospitalization and population-based in-hospital death rates in every age group aged ≥40 years. The distinction between the Southeast’s larger absolute burden and the South’s higher rates was therefore also evident within age strata, although these comparisons do not constitute age standardization or adjustment for clinical case mix. Research on disparities in pancreatitis care indicates that socioeconomic conditions, availability of healthcare resources, timely referral, and access to specialized services can affect clinical outcomes [17]. Accordingly, increasing hospitalization rates in several age groups in the North and Northeast may reflect changes in disease occurrence, diagnostic recognition, service availability, referral practices, or administrative reporting rather than a single underlying cause [4,9,17]. These increases were not uniform across older age groups, indicating that aggregate regional trends may obscure differences by age. Moreover, hospitalizations were classified according to the location of admission, whereas population denominators were based on the region of residence; consequently, cross-regional patient movement may have influenced regional estimates [9,10]. Regional differences in population-based in-hospital death rates therefore cannot be interpreted as direct measures of healthcare quality or individual mortality risk [17].

The demographic patterns observed in this study should also be considered in relation to established and potentially modifiable etiological factors. A meta-analysis of prospective studies identified elevated body mass index and central adiposity as factors associated with an increased risk of acute pancreatitis [18], while a systematic review documented a substantial prevalence of obesity among Brazilian adults [19]. Brazilian population-based studies have also demonstrated that harmful or heavy alcohol consumption is more common among men [20,21]. These exposures provide a plausible context for the observed sex-related differences because alcohol-associated pancreatitis and chronic pancreatitis occur more frequently among men, whereas gallstone-associated acute pancreatitis is more common among women [5,22]. The comparatively greater increase in hospitalizations among women may be compatible with changes in biliary or metabolic risk profiles; however, this interpretation remains speculative because information on gallstones, alcohol consumption, body mass index, serum triglyceride levels, and disease etiology was unavailable [5,9,18-22].

The largest cumulative admission counts occurred among adults aged 30-39 years in the North and Northeast, 40-49 years in the Southeast and Central-West, and 50-59 years in the South, whereas the highest age-specific hospitalization rates occurred among those aged ≥80 years in all five regions in 2024. This distinction indicates that the age groups generating the largest numbers of admission records were not necessarily those with the highest population-based rates. Adults aged ≥60 years accounted for at least half of recorded deaths in every region, with the greatest concentration in the South; nevertheless, the Central-West had its largest single age-group death count among those aged 50-59 years. The predominance of deaths at older ages is consistent with evidence linking advanced age to greater disease severity and mortality in acute pancreatitis [6]. A prospective cohort study further demonstrated that age-related outcomes should be interpreted in conjunction with the burden of comorbidities, which contributes to complications and mortality [23]. Population-based research involving older patients also identified substantial mortality and recurrence following an initial episode of acute pancreatitis, highlighting vulnerability beyond the index hospitalization [24]. Frailty constitutes an additional relevant determinant because a nationwide cohort study associated frailty with increased mortality, more frequent local and systemic complications, and greater healthcare resource utilization [25]. In conjunction with this external evidence, the observed concentration of deaths among older adults highlights the relevance of assessing comorbidities, frailty, organ dysfunction, and individualized treatment needs; however, these factors and the effects of their management were not evaluated in this study [6,23-25].

The combination of increasing admissions, relatively stable in-hospital death counts, and declining deaths relative to approved AIHs is consistent with patterns described in large inpatient studies from the United States [12,13]. In this study, the declining AIH-based death proportion reflects the increase in admission volume alongside the comparatively small change in recorded deaths; it does not independently establish improved clinical outcomes among comparable patients. Advances in clinical assessment, supportive care, nutritional management, etiological investigation, and multidisciplinary treatment offer one possible explanation for declining in-hospital mortality indicators [1]. Contemporary American College of Gastroenterology guidelines recommend appropriate fluid resuscitation, early oral or enteral nutrition, systematic evaluation of biliary and other etiologies, and evidence-based management of complicated acute pancreatitis [26]. However, the available administrative data do not establish whether these practices were implemented or contributed to the observed trend. Alternative explanations include changes in hospitalization thresholds, disease severity, the relative composition of K85-K86 diagnoses, case ascertainment, diagnostic coding, and access to hospital care [9,12,13]. Severe acute pancreatitis continues to be associated with substantial mortality among patients requiring intensive care, and reductions in late mortality do not necessarily translate into comparable improvements in early mortality [27]. Moreover, a population-based cohort demonstrated persistently elevated mortality after hospital discharge, indicating that in-hospital deaths capture only part of the overall disease burden [28]. Consequently, regional variations in AIH-based death proportions require consideration of disease severity, referral practices, access to specialized care, and longer-term outcomes before clinical or healthcare-quality explanations can be advanced [17,26-28].

The geographical and demographic differences identified in this study may inform the assessment of regional healthcare needs and priorities within SUS-funded care [4,8,17]. Timely diagnosis, coordinated referral pathways, access to specialized services, and adherence to evidence-based management are relevant considerations for patients with severe or complicated disease [1,17,26]. These are potential public health implications of the observed variation, rather than interventions whose effectiveness or local availability was evaluated by this study. Standardized etiological assessment and interventions targeting gallstone-related disease, harmful alcohol consumption, and metabolic risk factors may help prevent recurrent episodes and potentially avoidable hospitalizations [2,5,18-22,26]. Older adults may particularly benefit from systematic evaluation of comorbidities and frailty, individualized treatment planning, and appropriate post-discharge follow-up [6,23-25,28]. Future surveillance should distinguish acute pancreatitis, chronic pancreatitis, and other pancreatic disorders and incorporate age-standardized, sex-specific, and region-specific indicators to improve epidemiological comparability [2-4,9-11].

This study has limitations inherent to its ecological design and use of administrative hospital records, which preclude individual-level inference and causal attribution [7-9]. SIH/SUS captures SUS-funded admissions in public and contracted private hospitals but excludes admissions financed exclusively by private insurance or out-of-pocket payments, limiting generalizability to all hospital care in Brazil [8,9]. Approved AIHs represent administrative records rather than unique patients; repeated admissions, transfers, and authorizations associated with prolonged stays could not be linked. AIH-based rates therefore describe recorded hospital utilization rather than disease incidence or the number of affected individuals. Coding errors, diagnostic misclassification, reimbursement practices, service availability, and reporting completeness may influence observed patterns. Although aggregate consistency was checked, the underlying records could not be independently validated against clinical documentation [9]. Combining ICD-10 categories K85-K86 encompasses conditions with different etiologies, severity, treatment, and prognosis, and changes in their relative composition may affect comparisons [1,2,9]. Regional numerators were assigned by hospital location, whereas denominators represented resident populations; interregional referrals may therefore influence regional rates. Age-specific analyses supported comparisons within corresponding age groups but did not age-standardize overall rates or eliminate residual differences within broad age categories [3,4,6]. The absence of information on clinical severity, comorbidities, etiology, and treatment prevented adjustment for case mix. Mortality indicators captured only recorded in-hospital deaths, excluding deaths before admission or after discharge; deaths relative to approved AIHs therefore do not estimate case fatality among unique patients or longer-term survival [9,23,25-28]. Furthermore, only 10 annual observations were available for each Joinpoint model, limiting the stability of trend estimates and the detection of complex temporal patterns. Increased admission rates cannot establish increased disease incidence, and declining deaths relative to AIHs cannot establish improved care; explanations involving pandemic-related disruptions, changes in access, or improvements in management remain hypotheses. Despite these limitations, the study provides nationwide evidence on SUS-funded hospital utilization over a decade encompassing the COVID-19 pandemic and subsequent recovery. Its strengths include the large administrative dataset, complementary admission and mortality indicators, regional and demographic stratification, and Joinpoint analyses extending beyond comparisons between the first and last study years. Reconciliation of totals and independent verification of rate calculations further strengthened internal consistency.

## Conclusions

SUS-funded emergency admission rates under the combined ICD-10 categories K85-K86 increased in Brazil between 2015 and 2024, while recorded in-hospital death counts remained relatively stable and deaths relative to approved Hospital Admission Authorizations (AIHs) declined. These findings describe administrative admission records rather than unique patients and do not establish increased disease incidence or improved clinical care. The temporary decline in admissions in 2021 and subsequent increase coincided with the pandemic and following years; disruptions in access and changes in hospital demand remain possible explanations rather than demonstrated mechanisms.

Regional differences were evident in absolute admission and death counts, population-based rates, and temporal trends. The Southeast accounted for the largest absolute burden, whereas the South presented the highest crude population-based admission and in-hospital death rates. Age-specific analyses provided additional detail but did not eliminate demographic confounding in overall comparisons. Male predominance and the concentration of deaths among older adults further characterized the distribution of recorded hospital burden. These patterns should be interpreted alongside differences in population structure, referral flows, and administrative recording, rather than as direct measures of individual risk or healthcare quality.

The observed variation may inform assessments of regional emergency capacity, referral pathways, and access to specialized pancreatic care. Prevention of recurrent disease, etiological investigation, and assessment of clinical vulnerability are relevant considerations supported by the broader literature, although their effects were not evaluated in this study. Future surveillance incorporating separate diagnostic categories, age-standardized indicators, and linked clinical and post-discharge information could help clarify these patterns and support more equitable healthcare planning.
